# Severe acute respiratory coronavirus virus 2 (SARS-CoV-2) seroprevalence in healthcare personnel in northern California early in the coronavirus disease 2019 (COVID-19) pandemic

**DOI:** 10.1017/ice.2020.1358

**Published:** 2020-12-09

**Authors:** Joelle I. Rosser, Katharina Röltgen, Melissa Dymock, John Shepard, Andrew Martin, Catherine A. Hogan, Andra Blomkalns, Roshni Mathew, Julie Parsonnet, Benjamin A. Pinsky, Yvonne A. Maldonado, Scott D. Boyd, Sang-ick Chang, Marisa Holubar

**Affiliations:** 1 Division of Infectious Diseases & Geographic Medicine, Department of Medicine, Stanford University School of Medicine, Stanford, California; 2 Department of Epidemiology and Population Health, Stanford University School of Medicine, Stanford, California; 3 Department of Pathology, Stanford University School of Medicine, Stanford, California; 4 Stanford Health Care, Stanford, California; 5 Department of Emergency Medicine, Stanford University School of Medicine, Stanford, California; 6 Division of Infectious Diseases, Department of Pediatrics, Stanford University School of Medicine, Stanford, California

## Abstract

**Objective:**

We assessed the magnitude of unidentified coronavirus disease 2019 (COVID-19) in our healthcare personnel (HCP) early in the COVID-19 pandemic, and we evaluated risk factors for infection to identify areas for improvement in infection control practice in a northern California academic medical center.

**Methods:**

We reviewed anti–severe acute respiratory coronavirus virus 2 (SARS-CoV-2) receptor-binding domain (RBD) IgG serologic test results and self-reported risk factors for seropositivity among 10,449 asymptomatic HCP who underwent voluntary serology testing between April 20 and May 20, 2020.

**Results:**

In total, 136 employees (1.3%) tested positive for SARS-CoV-2 IgG. This included 41 individuals (30.1%) who had previously tested positive for SARS-CoV-2 by nasopharyngeal reverse-transcription polymerase chain reaction (RT-PCR) between March 13 and April 16, 2020. In multivariable analysis, employees of Hispanic ethnicity (odds ratio [OR], 2.01; 95% confidence interval [CI], 1.22–3.46) and those working in environmental services, food services, or patient transport (OR, 4.81; 95% CI, 2.08–10.30) were at increased risk for seropositivity compared to other groups. Employees reporting a household contact with COVID-19 were also at higher risk for seropositivity (OR, 3.25; 95% CI, 1.47–6.44), but those with a work, exposure alone were not (OR, 1.27; 95% CI, 0.58–2.47). Importantly, one-third of seropositive individuals reported no prior symptoms, no suspected exposures, and no prior positive RT-PCR test.

**Conclusion:**

In this study, SARS-CoV-2 seropositivity among HCP early in the northern California epidemic appeared to be quite low and was more likely attributable to community rather than occupational exposure.

Severe acute respiratory coronavirus virus 2 (SARS-CoV-2) has rapidly spread around the world presenting as asymptomatic coronavirus disease 2019 (COVID-19) to life-threatening respiratory and multiorgan system failure. The rate of infection of HCP has been extremely high in some settings, and work exposure is a significant concern.^1-3^ Infections among healthcare personnel (HCP) pose a threat not only to HCP themselves but also to the patients they care for and to their coworkers.^4^ In addition, the impact on the health system from losing HCP to quarantine, illness, or death jeopardizes its ability to provide essential medical care, particularly when confronting a surge of critically ill patients. Nosocomial transmission to patients is particularly worrisome because hospitalized patients frequently have comorbidities that put them at increased risk of poor outcomes from COVID-19. These concerns have given rise to a call for aggressive testing of HCP to identify both symptomatic and asymptomatic infection.^5,6^

The San Francisco Bay Area, a metropolitan area with a large population of international travelers, had early exposure to SARS-CoV-2–infected individuals, with several of the first cases going unrecognized. Santa Clara County identified its first case of community-acquired COVID-19 on February 27, 2020, and retrospective autopsy reports identified cases as early as February 6.^7,8^ At Stanford Healthcare, a retrospective study conducted on nasopharyngeal samples initially ordered to test for other viral respiratory pathogens from symptomatic individuals detected SARS-CoV-2 starting as early as February 21, 2020.^9^ In 4 urgent-care clinics in Santa Clara County, 11% of patients with influenza-like illness between March 5 and 14, 2020, who tested negative for influenza tested positive for SARS-CoV-2.^7^ Given this evidence of unrecognized community transmission, Bay Area HCP could have been unknowingly exposed to SARS-CoV-2 early in the epidemic.

In April and May of 2020, Stanford Healthcare offered voluntary serologic screening for anti–SARS-CoV-2 RBD IgG antibodies to all of its HCP. By the start of HCP serologic screening, >1,100 outpatients and >300 inpatients at Stanford had been diagnosed with SARS-CoV-2.^10^ We conducted a quality improvement and quality assurance study using HCP serologies to estimate the magnitude of undiagnosed COVID-19 in our HCP early in the epidemic and to assess self-reported occupational and household risk factors for seropositivity.

## Methods

### Study design and oversight

We conducted a review of 10,449 asymptomatic employees who were surveyed and tested for evidence of SARS-CoV-2 infection by serology through an institutional initiative between April 20 and May 20, 2020. Participation in the testing was strictly voluntary. Stanford Healthcare is an academic medical health system that includes a university hospital, a children’s hospital, and multiple clinics as well as a network of affiliated community hospitals and clinics. The Stanford Healthcare Institutional Review Board approved this quality improvement and quality assurance study.

### Inclusion and exclusion criteria

All of the asymptomatic employees who underwent voluntary serologic testing and completed an online survey administered as a part of this initiative between April 20 and May 20, 2020, were included in the analysis. Samples from anyone who reported symptoms at the time of serologic testing or did not complete the survey were excluded from the analysis.

### Data collection

Survey data were directly entered by employees into the Stanford-hosted REDCap data management tool at the time of serologic testing.^11,12^ The survey asked if respondents had (1) a suspected work exposure to a patient or colleague with COVID-19 for which they were notified by occupational health, or a suspected work exposure (to patients or to colleagues) that they self-identified, (2) a suspected or confirmed exposure to household contact with COVID-19, or (3) a nonwork or nonhome exposure that they self-identified. Individuals were categorized into 1 of 4 mutually exclusive categories: (1) responding yes to a work exposure only, (2) responding yes to a household exposure only, (3) responding yes to both work and household exposures, and (4) responding yes to a nonwork or nonhousehold exposure only. Individuals reporting a household exposure were further asked about the testing status of the contact and whether the contact was a child. Respondents were also prompted to select from a list of COVID-associated symptoms that they had at the time of serologic screening and in the preceding months as well as the timing of these symptoms.

### Laboratory testing

All laboratory data were ordered as part of occupational health clinical services. Laboratory test results were extracted from the electronic medical record system (EMR; EpicSystems, Verona, WI). Serological testing was conducted in the Stanford Clinical Laboratory using their emergency-use–authorized anti–SARS-CoV-2 RBD enzyme-linked immunosorbent assay (ELISA). Validation of the SARS-CoV-2 RBD IgG assay in the Stanford Clinical Laboratory for CLIA testing documentation showed that the assay has a sensitivity of 98.1% for RT-PCR positive inpatients and 87.0% for asymptomatic RT-PCR positive individuals and symptomatic positive outpatients in the fourth week after symptom onset. Sensitivity may be lower earlier or later in the disease course.^13^ The SARS-CoV-2 RBD IgG serology assay was performed as described by Röltgen et al^13^ and had a specificity of 99.75% determined by testing 397 prepandemic samples. Individuals were considered to be seropositive if they had a positive anti–SARS-CoV-2 RBD IgG test result on the day the survey was completed. Individuals were considered to have had a prior positive RT-PCR result if they had a documented positive RT-PCR performed at the Stanford Clinical Laboratory any time prior to the day of serologic testing. RT-PCR tests performed outside the Stanford system or performed on the same day as serologic testing were not included.

### Data analysis

Seropositivity rates are described for demographic and work characteristics, and predictors of seropositivity were evaluated using univariate and multivariable analyses. Univariate analyses were performed using the Fisher exact test and logistic regression. Multivariable analysis was performed using logistic regression. All risk factors for seropositivity with a *P* value of <.10 on univariate analysis as well as interaction terms were added to the model in a forward stepwise manner. The final model was selected based on the best fit as determined by the lowest Akaike information criterion (AIC). All statistical analyses were performed using R version 1.1.456 software (R Foundation for Statistical Computing, Vienna, Austria).

## Results

### Demographics

In total, 11,330 HCP completed the survey and underwent serology testing through occupational health services between April 20 and May 20, 2020. Among them, 881 individuals were excluded because they reported symptoms at the time of testing, leaving 10,449 subjects in the final analysis. Respondents primarily consisted of nurses (40.4%) and physicians (19.9%) but also included various patient support staff ranging from physical therapists to environmental services professionals and administrators (Table 1).

**Table 1.** Demographic and Work Characteristics

Characteristics	Seropositive (N=136), No. (%)	Seronegative (N=10,313), No. (%)	Odds Ratio (95% CI)^a^
**Age, y**			
Mean (SD)	39.0 (11.6)	40.9 (11.6)	0.99 (0.97–1.00)
Range	21.0–68.0	18.0–84.0	
**Sex**			
Female	81 (66.9)	6,709 (70.0)	Reference
Male	40 (33.1)	2,874 (30.0)	1.15 (0.78–1.68)
Unknown	15	730	
**Ethnicity**			
Non-Hispanic	59 (70.2)	5,342 (86.3)	Reference
Hispanic	25 (29.8)	846 (13.7)	**2.68 (1.64–4.24)**
Unknown	52	4125	
**Race**			
White	41 (62.1)	2,945 (57.1)	Reference
Asian	20 (30.3)	1,770 (34.3)	0.81 (0.46–1.37)
Black	4 (6.1)	204 (4.0)	1.41 (0.42–3.53)
Other	1 (1.5)	241 (4.7)	0.30 (0.02–1.38)
Unknown	70	5153	
**Household size**			
Mean (SD)	2.9 (1.6)	3.00 (1.5)	0.97 (0.87–1.09)
Range	1.0–7.0	1.0–7.0	
No response	3	143	
**County of residence**			
Santa Clara	58 (44.3)	4,514 (44.4)	Reference
San Mateo	37 (28.2)	2,376 (23.4)	1.21 (0.79–1.83)
Alameda	17 (13.0)	1,689 (16.6)	0.78 (0.44–1.32)
San Francisco	13 (9.9)	663 (6.5)	1.53 (0.8–2.71)
Other	6 (4.6)	916 (9.0)	0.51 (0.2–1.09)
No response	5	155	
**Work role**			
Nurse, medical assistant, phlebotomist	55 (40.4)	3,904 (37.9)	Reference
Physician, advanced practice provider, trainee	27 (19.9)	2,506 (24.3)	0.76 (0.47–1.2)
Environmental Services, food service, patient transport	12 (8.8)	323 (3.1)	**2.64 (1.33**–**4.8)**
Therapist (eg, physical, occupational, speech), tech	4 (2.9)	249 (2.4)	1.14 (0.34–2.81)
Laboratory technician	1 (0.7)	224 (2.2)	0.32 (0.02–1.45)
Unit clerk	0 (0.0)	218 (2.1)	0.00 (0–0.66)
Pharmacist or pharmacy technician	4 (2.9)	209 (2.0)	1.36 (0.41–3.35)
Respiratory therapist	0 (0.0)	135 (1.3)	0.00 (0–28.41)
Social worker or interpreter	2 (1.5)	115 (1.1)	1.23 (0.2–4.03)
Research	1 (0.7)	101 (1.0)	0.70 (0.04–3.24)
Administration	13 (9.6)	959 (9.3)	0.96 (0.5–1.71)
Other	17 (12.5)	1,370 (13.3)	0.88 (0.49–1.49)
**Primary work setting**			
Inpatient unit	29 (21.3)	2,480 (24.0)	Reference
Outpatient specialty clinic	19 (14.0)	1,927 (18.7)	0.84 (0.46–1.5)
Intensive care unit	13 (9.6)	952 (9.2)	1.17 (0.58–2.21)
Operating room	11 (8.1)	884 (8.6)	1.06 (0.51–2.08)
Administrative office	14 (10.3)	655 (6.4)	1.83 (0.93–3.42)
Outpatient primary care clinic	3 (2.2)	551 (5.3)	0.47 (0.11–1.32)
Emergency department	8 (5.9)	539 (5.2)	1.27 (0.54–2.66)
Radiology	6 (4.4)	407 (3.9)	1.26 (0.47–2.85)
Laboratory	5 (3.7)	389 (3.8)	1.10 (0.37–2.62)
Other	28 (20.6)	1,529 (14.8)	1.57 (0.92–2.65)
Also works at an outside healthcare facility	13 (9.6)	1,578 (15.3)	0.59 (0.31–1)
**Works directly with respiratory secretions (Physicians, Nurses, RT only)**			
No	64 (78.0)	5,498 (84.0)	Reference
Yes	18 (22.0)	1,047 (16.0)	1.48 (0.85–2.45)
No response	54	3768	
**SARS-CoV-2 RT-PCR results prior to serologic testing**			
Positive RT-PCR test	41 (30.1)	4 (0.0)	
Negative RT-PCR test	13 (9.6)	1,127 (10.9)	
Not tested at Stanford prior to serologic testing	82 (60.3)	9,182 (89.0)	

Note. CI, confidence interval; SD, standard deviation; RT, respiratory therapist; RT-PCR, reverse transcription polymerase chain reaction.

a SARS-CoV-2 IgG seropositivity rates for different demographic and work groups were compared by univariate logistic regression. Characteristics significantly associated with seropositivity are indicated in bold.

Of 10,449 employees, 136 (1.3%) had a positive SARS-CoV-2 RBD IgG serology. Among these 136 individuals, 41 (30%) had a prior positive RT-PCR confirmed by the Stanford Laboratory records system, 13 (10%) had a prior negative RT-PCR in our system, and 82 (60%) had no prior RT-PCR result in our system. Therefore, the overall rate of possible undiagnosed COVID-19 cases was 95 of 10,449 (0.9%). Individuals of Hispanic ethnicity were more likely than those of non-Hispanic ethnicity to have a seropositive test (odds ratio [OR], 2.68; *P* < .001). Individuals working in environmental services, food service, or patient transport also had significantly higher odds of having a positive serology compared to the reference group (OR, 2.64; *P* < .001). No other demographic or work characteristics were significantly associated with seropositivity (Table 1).

### Reported exposures

A possible COVID-19 exposure was reported by 2,105 of 10,449 individuals (20.1%), and this exposure was associated with a 3.10 higher odds of seropositivity (95% confidence interval [CI], 2.16–4.42) compared to individuals with no reported exposure. Employees with any reported exposure were also more likely to have had a prior RT-PCR test performed in our laboratory (OR, 3.13; 95% CI, 2.75–3.57). Both before and after removing individuals with a prior positive RT-PCR test, reported exposure was associated with an increased odds of seropositivity, except for those who reported a possible work exposure only. Similarly, among individuals with a prior RT-PCR test, individuals who reported a possible exposure were more likely to have a positive RT-PCR test result except for those who reported a possible work exposure only (Table 2).

**Table 2.** Reported Exposure to COVID-19 Prior to Serologic Testing

Exposure Category^a^	No. (%) of Responses	Seropositivity				RT-PCR Test Positivity Prior to Serologic Testing^d^	
		Overall^b^		Individuals without a Prior Positive RT-PCR Test^c^			
		No. (%)	Odds Ratio (95% CI)^e^	No.(%)	Odds Ratio (95% CI)^e^	No. (%)	Odds Ratio (95% CI)^e^
No Reported Exposure	8,344 (79.9)	77/8,344 (0.9)	Reference	67/8,333 (0.8)	Reference	11/710 (1.5)	Reference
Work Exposure ONLY	1,314 (12.6)	20/1,314 (1.5)	1.66 (0.98–2.67)	12/1,306 (0.9)	1.71 (0.66–4.27)	8/305 (2.6)	1.71 (0.66–4.27)
Household Exposure ONLY	490 (4.7)	17/490 (3.5)	**3.86** **(2.19–6.41)**	6/477 (1.3)	**11.32** **(4.89–26.66)**	13/86 (15.1)	**11.32** **(4.89–26.66)**
BOTH Work and Household Exposure	136 (1.3)	12/136 (8.8)	**10.39** **(5.26–18.88)**	6/129 (4.7)	**9.67** **(3.42–25.76)**	7/53 (13.2)	**9.67** **(3.42–25.76)**
Non-work/Non-household Exposure	165 (1.6)	10/165 (6.1)	**6.93** **(3.31–13.03)**	4/159 (2.5)	**15.25** **(4.93–43.59)**	6/31 (19.4)	**15.25** **(4.93–43.59)**
Overall		136/10,449 (1.3)		95/10,404 (0.9)		45/1,185 (3.8)	

Note. RT-PCR, reverse transcription polymerase chain reaction; CI, confidence interval.

a All individuals were categorized into one of five categories based on their suspected reported COVID-19 exposures (column 1 and 2).

b Across all individuals, seropositivity was higher compared to no reported exposure for all of the exposure groups, except ‘Work Exposure ONLY’ (columns 3 and 4).

c Excluding individuals with a known positive RT-PCR test prior to serologic testing, the odds of seropositivity remain higher compared to no reported exposure for all of the exposure groups, except ‘Work Exposure ONLY’(columns 5 and 6).

d RT-PCR test results included all SARS-CoV-2 RT-PCR tests performed at Stanford before the individual’s serologic screening between April 20 and May 20, 2020. All positive RT-PCR tests were performed between March 14 and April 16, 2020. Among individuals who had a RT-PCR test performed prior to serology, RT-PCR test positivity rates are again higher for all of the exposure groups, except “Work Exposure ONLY” (columns 7 and 8).

e Odds ratios represent the results of univariate logistic regression. Significant results are indicated in bold.

f Work exposure includes exposures confirmed by occupational health and presumed work exposures (not notified by occupational health) and includes exposure to patients or colleagues.

A suspected household contact was reported by 29 (21.3%) of the seropositive individuals and by 597 (5.8%) of the seronegative individuals. Of these 626 individuals reporting a suspected household contact, 35 (5.6%) reported that the household contact was confirmed to have COVID-19 with a positive RT-PCR test, 57 (9.1%) reported the contact had a negative RT-PCR test, and 534 (85.3%) reported no prior RT-PCR testing. Seropositivity of employees was significantly associated with reporting that the household contact was confirmed by a positive RT-PCR test (34.5% vs 4.2%; OR, 11.92; 95% CI, 4.47–30.47). Of individuals with a reported household contact, seropositive individuals were less likely to report that the contact was a child (2 of 29, 6.9%) compared to seronegative individuals (128 of 469, 21.4%), although this factor did not reach statistical significance (OR, 0.27; 95% CI, 0.03–1.11).

### Multivariable analysis

The final multivariable regression model of risk factors for seropositivity included ethnicity, work role, and exposure category. The final model included 6,272 observations (60.0%) because ethnicity was unknown for the remaining observations. All demographic and risk groups were well represented in the final model. Notably, the final model included at least 43% of each work category including 51% of HCP in environmental services, food services, or patient transport. Among individuals reporting ethnicity, Hispanic ethnicity closely correlated with working in environmental services, food services, or patient transport (50.6%) compared to working in all other roles (12.9%). Including an interaction term between ethnicity and working in environmental services, food services, or patient transport in the multivariable model did not improve model fit and was therefore not included in the final model. Hispanic ethnicity (OR, 2.01; 95% CI, 1.22–3.46) and working in environmental services, food service, or patient transport (OR, 4.81; 95% CI, 2.08–10.30) both remained significantly associated with seropositivity in the multivariable model. Similar to the univariate analysis, reporting a COVID-19 work exposure only was not significantly associated with seropositivity after adjusting for ethnicity and work role, but the suspected nonwork or nonhousehold exposure as well as suspected household exposure with or without work exposure categories remained significant predictors of seropositivity (Table 3).

**Table 3.** Multivariable Regression^a^

Characteristic	Odds Ratio (95% CI)^b^
**Ethnicity**	
Non-Hispanic	Reference
Hispanic	**2.09 (1.22–3.46)**
**Work role**	
Nurse, MA, or phlebotomist	Reference
Physician, APP, or trainee	0.91 (0.49–1.65)
Environmental services, food service, patient transport	**4.81 (2.08–10.39)**
Therapist or Tech	1.83 (0.43–5.34)
Laboratory technician	0.65 (0.04–3.17)
Unit clerk	0 (0–60.32)
Pharmacist or pharmacy technician	2.16 (0.50–6.34)
Respiratory therapist	0 (0 to >100)
Social worker or interpreter	1.23 (0.07–6.00)
Research	2.42 (0.13–12.07)
Administration	1.23 (0.54–2.57)
Other	1.05 (0.48–2.13)
**Reported COVID-19 exposures**	
No reported exposure	Reference
Work exposure ONLY	1.27 (0.58–2.47)
Household exposure ONLY	**3.25 (1.47–6.44)**
Both work and household exposure	**13.83 (6.27–27.98)**
Nonwork, nonhousehold exposure	**7.03 (2.37–16.79)**

Note. CI, confidence interval.

a The final multivariable regression model above included 6,272 (60.0%) of the original 10,449 observations due to missing ethnicity data.

b Significant associations are indicated in bold.

### Prior symptoms

In total, 3,422 (32.7%) individuals reported having any symptoms associated with COVID-19 since February 1, 2020; of these, only 41 (1.2%) were seropositive. Those with previous symptoms were significantly more likely to have had prior RT-PCR testing: 839 of 3,422 (24.5%) of previously symptomatic people had a RT-PCR test result versus only 346 of 7,027 (4.9%) of individuals who reported no previous symptoms (OR, 6.27; 95% CI, 5.48–7.19). Having any prior symptoms was significantly associated with seropositivity overall: 74 of 3,422 (2.2%) versus 62 of 7.027 (0.9%) (OR, 2.48; 95% CI, 1.74–3.55). Evaluating only individuals without a previous positive RT-PCR test was also significantly associated with seropositivity overall: 41 of 3,385 (1.2%) versus 54 of 7,019 (0.8%) (OR = 1.58; 95% CI, 1.03–2.42). Fever or chills, muscle aches, and symptoms identified by participants as ‘other’ had the strongest correlation with seropositivity. Additionally, 1,189 people (11.4%) with no prior positive RT-PCR test thought they had COVID-19 in the months prior to serologic testing. Only 28 of 1,189 (2.3%) were seropositive, but having this belief was associated with seropositivity (OR, 3.29; 95% CI, 2.03–5.21) (Table 4).

**Table 4.** Reported Symptoms Prior to Serology Testing

Symptom	Seropositive (N=95), No. (%)	Seronegative (N=10,309), No. (%)	Odds Ratio (95% CI)^a^
Any symptoms	41 (43.2)	3,344 (32.4)	**1.58 (1.03–2.42)**
Congestion	19 (20.0)	1,673 (16.2)	1.29 (0.73–2.16)
Sore throat	18 (18.9)	1,615 (15.7)	1.26 (0.71–2.13)
Cough or shortness of breath	22 (23.2)	1,506 (14.6)	**1.76 (1.04–2.13)**
Headache	21 (22.1)	1,407 (13.6)	**1.80 (1.05–3.00)**
Fatigue	20 (21.1)	1,209 (11.7)	**2.01 (1.16–3.34)**
Fever or chills	19 (20.0)	910 (8.8)	**2.58 (1.47–4.34)**
Muscle aches	20 (21.1)	899 (8.7)	**2.79 (1.61–4.65)**
Nausea, vomiting, diarrhea	7 (7.4)	571 (5.5)	1.36 (0.53–2.93)
Other symptoms	5 (5.3)	82 (0.8)	**6.92 (2.14–17.43)**
Thought they had COVID-19 prior to serologic testing	28 (29.5)	1,161 (11.3)	**3.29 (2.03–5.21)**

Note. This table includes the exact list of symptoms asked of HCP in the survey. Individuals could select multiple symptoms. Prior symptoms included any symptoms between February 1, 2020 and the time of serologic testing in April or May 2020.

a Univariate analysis was performed using the Fisher exact test and significant results are indicated in bold.

Of all individuals reporting prior symptoms, onset was highest in February and early March; 28.6% (n = 1,006) reported early February, 19.0% (n = 666) reported late February, 28.2% (n = 992) reported early March, 14.2% (n = 497) reported late March, 7.5% (n = 265) reported early April, and 2.5% (n = 86) reported late April. Seropositivity was highest in people reporting first symptoms in March and lowest in February. Seropositivity by date of symptom onset was as follows: 10 of 1,006 (1.0%) in early February; 3 of 666 (0.5%) in late February; 33 of 992 (3.3%) in early March; 22 of 497 (4.4%) in late March; 5 of 265 (1.9%) in early April; and 2 of 86 (2.3%) in late April.

Almost half of seropositive individuals reported no prior symptoms (62 of 136, 45.6%), more than half had no suspected exposure (77 of 136, 56.6%), and more than two-thirds had either a negative prior test (13 of 136, 9.6%) or had not previously been tested (82 of 136, 60.3%). In total, 46 seropositive individuals (33.8%) had none of these indicators: prior symptoms, suspected exposures, or positive RT-PCR tests.

## Discussion

Our study of >10,000 HCP at a major medical center shows a low seroprevalence overall. Importantly, our analysis also indicated that hospital exposure alone was not a major risk for transmission and was not associated with seropositivity for SARS-CoV-2 early in the COVID-19 pandemic. Although background community seroprevalence was unknown at this time for a direct comparison, our analysis suggests that community transmission appeared to be a more likely source of infections amongst HCP. These findings are consistent with other studies, demonstrating that healthcare worker infection rates reflect community transmission rates.^14^ Importantly, reported work exposure alone was not a risk factor for seropositivity or increased RT-PCR positivity rates. In contrast, having a household exposure (with or without a work exposure) or having a nonwork or nonhousehold exposure was significantly associated with both seropositivity and previously testing positive by RT-PCR. We also detected a strong association between Hispanic ethnicity and seropositivity. This finding is consistent with the increased risk of COVID-19 infection among Hispanics based on data from California public health records broadly as well as from Santa Clara and San Mateo Counties, the 2 counties where most of the HCP included in this study reside.^15-17^ Our study also shows a peak seropositivity in individuals reporting onset of symptoms in late March. This matches the epidemic curves in Santa Clara and San Mateo Counties in which infections peaked in late March followed by a decline in April and May.^16,17^

A strength of this study is the inclusion of HCP with varying roles and degrees of patient contact. Given the limited data on community seroprevalence at this early part of the pandemic, we were able to assess differences in presumed nosocomial infection rates among staff with presumably very high risk (eg, nurses and respiratory therapists) and those with a risk level comparable to the general community (eg, administrators). The only work group associated with a higher risk of seropositivity included individuals working in environmental services, food services, or patient transport. The reason for this finding is unclear. Possible explanations include an unrecognized risk associated with these jobs, inadequate personal protective equipment use due to either decreased awareness or perception of risk or lack of training, or more likely sociodemographic differences in risk for household or community exposure among workers in these occupations. We did adjust for ethnicity and reported exposures, and we tested for significant interactions in multivariable analysis to account for these additional risk factors, and this association remained significant.

Our findings have reinforced evolving SARS-CoV-2 policies within our healthcare system. In response to changing community prevalence and public health recommendations, our infection prevention policies changed throughout the study period. Before the first documented case of COVID-19 in the United States, we implemented a personal protective equipment (PPE) policy consistent with Centers for Disease Control and Prevention (CDC) guidelines. All staff were trained in the proper use of PPE using multiple modalities, including online videos, posters, and in-person training which was reinforced by unit-level management. We implemented a mandatory attestation of the lack of symptoms at hospital entrances for all employees. In early April 2020, as the prevalence of COVID-19 in our community increased, we implemented universal procedure masks for all personnel on the medical campus interacting with patients, including those without direct patient care responsibilities, and we significantly limited visitors. In late April, we also began screening all asymptomatic preoperative patients with nasopharyngeal SARS-CoV2 RT-PCR. The findings from this study provide reassurance that these policies, among others, substantially limited hospital transmission of SARS-CoV-2 to our HCP. These findings have also reinforced the importance of community transmission and have supported more aggressive measures to identify and prevent transmission from asymptomatic patients and visitors. By the end of May following this initiative, we had expanded screening to all asymptomatic patients admitted to the hospital. Our universal masking policy was reinforced by this study and subsequently by California Department of Public Health recommendations for universal mask wearing in the community^18^. Our findings also highlight the importance of supporting individuals working in indirect patient contact roles (eg, food services, environmental services, or patient transport) and minority groups to minimize both hospital and community risks of transmission.

This study has several limitations. Our ability to fully differentiate between community and work risk factors was limited by several factors. As has been a limitation in many healthcare data sets across the United States,^19^ reporting rates on race and ethnicity, 2 important demographic characteristics, were low in our data set. We were also unable to describe exposure risk in any further detail given the limited questions included in the survey. However, other studies have also noted higher infection rates early in the epidemic in non-frontline HCP, consistent with our findings.^2,20^ The low overall seropositivity in the region where the study took place is also a limitation of this study. This low event rate may have limited our ability to accurately identify meaningful associations. Additionally, low prevalence may increase the possibility of false positives, although the specificity of the SARS-CoV-2 RBD IgG assay used was high (99.75%), with only 1 in 397 prepandemic specimens tested yielding a false-positive result.^13^

In summary, the findings of this study in a nonsurge, well-resourced hospital in California with no PPE shortages and substantial access to occupational testing demonstrate low rates of undiagnosed HCP SARS-CoV-2 infection early in the epidemic. It also shows increased infection risk most likely attributable primarily to community spread. Infection risk in HCP can vary across settings based on dynamics of the local epidemic, availability of adequate PPE, and hospital capacity.^1^ Further work is needed to refine the occupational versus community predictors of COVID-19 risk in other settings and to determine the most effective ways to help HCP avoid not only hospital transmission but also community transmission of SARS-CoV-2.
